# Disseminated tuberculosis mimicking abdominal metastatic carcinoma: A case report

**DOI:** 10.1097/MD.0000000000027886

**Published:** 2021-11-24

**Authors:** Qi Zhou, MiaoXin Zhang

**Affiliations:** Department of Gastroenterology, Tongji Hospital, Tongji Medical College, Huazhong University of Science and Technology, Wuhan, Hubei, P.R. China.

**Keywords:** 18F-FDG PET/CT, EUS-FNA, extra-pulmonary tuberculosis

## Abstract

**Rationale:**

Extra-pulmonary tuberculosis (TB) has long been a diagnostic challenge for clinicians, often requiring extensive workup and invasive procedures, with the risk of significant complications. Endoscopic ultrasound-guided fine-needle aspiration (EUS-FNA) is a minimally invasive and highly accurate diagnostic modality for the evaluation of mediastinal and abdominal lymphadenopathy and masses. Several reports on the utility of EUS-FNA as a favorable method for diagnosing extrapulmonary TB have been published.

**Patient concerns:**

A 54-year-old man complained of intermittent melena.

**Diagnoses:**

18 fluorine-fluorodeoxyglucose positron emission tomography/computed tomography revealed suspected carcinoma metastasis. EUS-FNA did not reveal any evidence of malignancy.

**Interventions and outcomes:**

Laparoscopy was performed, and frozen section pathology during surgery showed granulomas with focal necrosis. Mycobacterium tuberculosis polymerase chain reaction was positive, but acid-fast bacilli staining was negative. Anti-TB treatment was initiated, and the patient was advised to visit the local TB dispensary regularly.

**Lessons:**

The presence of atypical inflammation of inadequate material or non-representative samples of extra-pulmonary TB was observed on EUS-FNA cytology. Mycobacterium tuberculosis polymerase chain reaction and acid fast bacilli should be performed to diagnose TB because of its higher sensitivity.

## 1 Introduction

Tuberculosis (TB) is one of the most common communicable diseases worldwide. According to the WHO Global Tuberculosis Report, in 2019, about 10 million people developed TB and 1.4 million died.^[1]^ While the lungs are the most common sites of TB, extrapulmonary TB has long been a diagnostic challenge for clinicians, which usually exhibits non-specific manifestations, often requiring extensive workup to obtain tissue samples and with the risk of delayed treatment.^[2–4]^ Herein, we report a case of disseminated abdominal TB that was mistakenly diagnosed as tumor metastasis. The patient underwent 18 fluorine-fluorodeoxyglucose positron emission tomography/computed tomography (18F-FDG PET/CT), endoscopic ultrasound-guided fine-needle aspiration (EUS-FNA), and laparoscopy.

## 2 Case presentation

A 54-year-old man was admitted to our hospital on October 31, 2019. He complained of intermittent melena for 8 months with abdominal pain and swelling. There was no fever, diarrhea, weakness, or vomiting. There was no history of comorbidities. The local hospital performed an esophagogastroduodenoscopy to reveal an ulcer (A1) in the corpus. The melena disappeared after medical treatment with a proton pump inhibitor but recurred after drug withdrawal. He was referred to our hospital for further management.

On physical examination, the lungs were clear, and middle quadrant abdominal pain was observed on deep palpation. No masses or enlarged lymph nodes were detected. Upon admission, laboratory investigations revealed mild anemia with a hemoglobin level of 110 g/L (reference 130–175 g/L). The lymphocyte count was 1.10 × 10^9^/L (reference 1.10–3.20 × 10^9^/L). Other laboratory investigations, including tumor markers, were normal. On radiographic examination, thoracic computed tomography revealed spotty calcification in the left upper lobe without specific signs of active or old pulmonary TB. The number of lymph nodes increased in the bilateral mediastinum and the armpits. Abdominal contrast-enhanced computed tomography revealed a protruding ulcerative mass in the greater curvature of the anterior gastric wall. Multiple soft tissue masses with localized calcification surrounded the gastric antrum, fundus, mesenteric vessels, and the sigmoid. In the ileum, there was segmental bowel wall thickening and mild enhancement, implying hemorrhage from terminal ileac diverticulitis. Possible carcinomas could not be ruled out in thin-section 1–11 paravertebral soft tissues. All the imaging findings showed a possibility of carcinoma or lymphoma.

**Figure 1:**
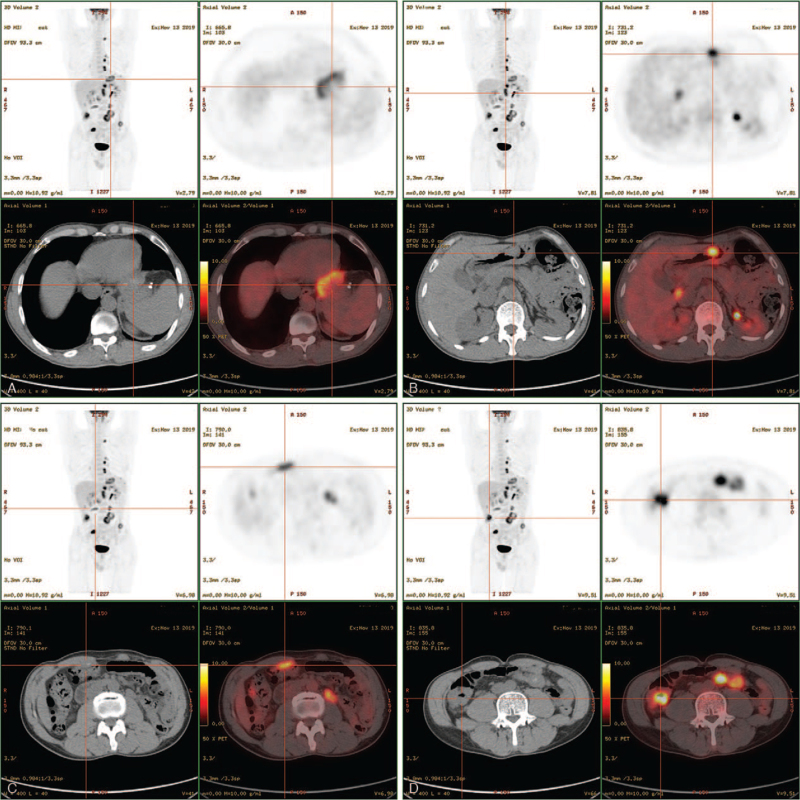
Increased FDG uptake under the diaphragm (A), in the corpus of the stomach (B), in the mesentery (C), and in the thickened parts of ileum and sigmoid (D).

To identify the nature of the lesions, the patient underwent 18F-FDG PET/CT on November 13, 2019. Increased FDG uptake was found in the left pleura, under the diaphragm (Fig. 1A), in the pelvis, hepatic-gastric (Fig. 3A) and porta-cava space around the stomach, abdominal aorta, and mesentery (Fig. 1C). Furthermore, FDG uptake was elevated in the thickened parts of the ileum and sigmoid (Fig. 1D) as well as in the gastric corpus (Fig. 1B). These results indicated a diagnosis of malignancy.

**Figure 2:**
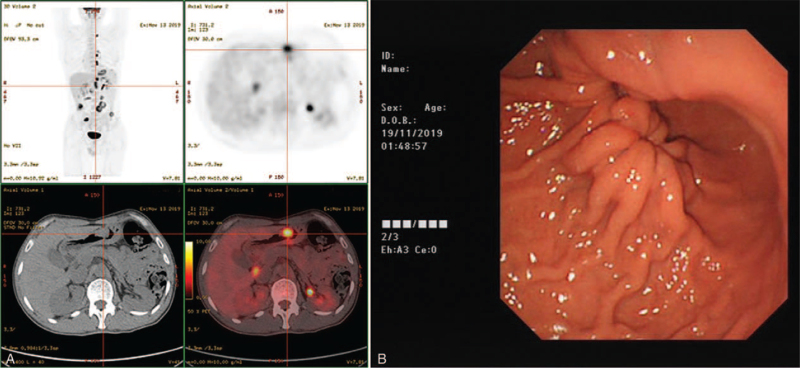
Gastric mucosa in the gastric corpus observed in gastroscopy (B) imaged as a FDG-avid mass (A) in 18F-FDG PET/CT.

**Figure 3:**
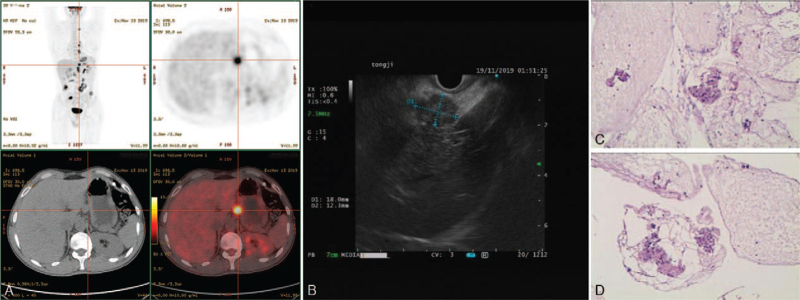
FDG-avid foci in the hepatic-gastric space (A); homogeneous hypoechoic lesion (18.0 mm × 12.3 mm) in the hepatic-gastric space (B); pathology of EUS-FNA tissue sample found scattered epithelial cells and spindle cells in the background of mucin. Immune markers: SMA(+), CD68(+), PCK(−), EMA(−), CK8/18(−), CD117(c-kit9.7)(−), CD34(−), CD117(positive control)(+), DOG1(−), DES(−), Caldesmon(−), S-100(−), SOX10(−), SDHB(−), Ki-67(L1 low proliferation) (C. H&E ×100; D. H&E ×200). EUS-FNA = endoscopic ultrasound-guided fine-needle aspiration.

EUS-FNA biopsy was performed to obtain a histological diagnosis in November 19, 2019. The gastric mucosa in the gastric corpus was observed macroscopically (Fig. 2B). Ultrasound endoscopy revealed a well-defined hypoechoic mass (20.7 mm × 8.6 mm) with homogeneous density originating from the fourth layer in the greater curvature of the corpus. A similar mass lesion (18.0 mm × 12.3 mm) (Fig. 3B) was seen in the hepatic-gastric space. Next to the neck of the pancreas, there was an enlarged retroperitoneal lymph node (18.8 mm × 22.0 mm). Repeated deep excavation biopsy of the gastric corpus and hepatic-gastric space showed scattered epithelial cells and inflammatory cells in the background of mucin with some adenoid structures. No malignant or atypical cells were observed. Immunohistochemistry was positive for SDHB, SMA, and CD68, together in favor of fibrous connective tissue (Fig. 3C and D).

**Figure 4:**
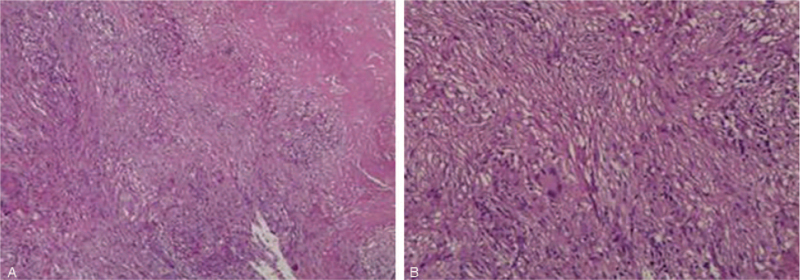
Part of the largest mass on the mesentery (about 8 cm × 8 cm) sent for frozen section pathology showed granulomas with focal necrosis. Molecular detection: TB RT- PCR(+), acid fast bacilli (AFB) staining(−). Immune markers: PCK(±), EMA(−), CK8/18(±), E-cad(−). (A. H&E ×40; B. H&E ×100).

To achieve a more adequate tissue sample, the patient underwent diagnostic laparoscopy. Intra-operatively there were multiple masses disseminated in the lesser curvature, diaphragmatic dome, pelvic cavity, lateral abdominal wall, mesentery, and small intestine wall. The largest one (approximately 8 cm × 8 cm) was located on the mesentery, which compressed the adjacent loops. Part of the largest mass was resected for frozen sections, which showed granulomas with focal necrosis (Fig. 4). Mycobacterium tuberculosis polymerase chain reaction (PCR) was positive, but acid-fast bacilli (AFB) staining was negative. Finally, a diagnosis of TB was established. Anti-TB treatment was initiated, and the patient was advised to visit the local TB dispensary regularly.

## 3 Patient consent

Informed consent was obtained from the patient for the purpose of publication of case details and images.

## 4 Discussion

Abdominal TB, which involves the gastrointestinal tract, peritoneum, lymph nodes, or solid organs, constitutes 12% of extra-pulmonary TB and 1% to 3% of total TB burden.^[5]^ The pathological characteristics include exudation, adhesion, and caseation. EUS-FNA has been confirmed as a safe, minimally invasive, and highly accurate diagnostic modality for mediastinal and abdominal lymphadenopathy and masses. The utility of EUS-FNA as a favorable method for diagnosing abdominal TB has been reported in several cases, but there is lack of studies with large samples, especially for patients with no ascites.^[6–11]^ Dhir et al^[5]^ enrolled in 66 patients with suspected abdominal TB for EUS-FNA cytology and only 7.6% of them had to undergo a surgical procedure to obtain a diagnosis. EUS-FNA has a risk of false-negative results due to inadequate sampling. It can be negative for TB and presents with atypical inflammation or without granulomas. Mohindra et al^[12]^ reported a higher sensitivity of Gene X-pert (97%) compared to cytology (77%) and AFB (39%) in EUS-FNA samples of 31 cases of abdominal TB. Sharma et al^[13]^ enrolled 4 case series for EUS-FNA of peritoneal nodules and concluded that the material should be sent for microbiological analysis if suspicion of TB is high. Therefore, for EUS-FNA, the initial examination of the specimen may not be diagnostic, and other tests such as AFB or Mycobacterium tuberculosis PCR are required. Taking histology as the gold standard, Mycobacterium tuberculosis PCR has a higher positive rate than AFB.^[14]^

Similar to previously reported cases,^[15–22]^ abdominal TB was not recognized based on imaging and was only identified on laparoscopy and biopsy. 18F-FDG PET/CT is widely performed to differentiate between benign and malignant diseases. A cohort study that compared PET/CT findings in TB peritonitis and peritoneal carcinoma showed similar FDG uptake for the 2 entities, with no significant difference in SUV max.^[23]^ TB lesions contain a large number of epithelioid cells, lymphocytes, and Langerhans cells that express high levels of glucose transporter 1 (Glut-1) and Glut-3, which induce high 18F-FDG uptake. This might be one of the main reasons that published studies have reported that TB peritonitis mimics peritoneal cancer on 18F-FDG PET/CT. Laparoscopy is invasive but useful for establishing a histological diagnosis, and frozen sections are useful for assessing the adequacy of biopsy and sampling.^[24,25]^

In our case, we overvalued the diagnostic accuracy of 18F-FDG PET/CT for malignant diseases. The cytology and pathology of EUS-FNA of the stomach and hepatic-gastric space did not show typical necrosis with granulomas for TB. Therefore, the differentiation of TB was not considered. We should realize the defects of the aforementioned 2 methods in the diagnosis of TB.

## 5 Conclusion

We present a case report of several diagnostic modalities to confirm the diagnosis of disseminated abdominal TB. This reminds us of the great challenge of differentiating extrapulmonary TB from malignant diseases. A high index of suspicion for TB is required.

## Author contributions

**Investigation:** Miaoxin Zhang.

**Resources:** Qi Zhou.

**Validation:** Qi Zhou.

**Writing – original draft:** Miaoxin Zhang.

**Writing – review & editing:** Qi Zhou.
